# Optimal threshold of time interval from symptom onset to diagnosis for identification of severity and outcomes in acute symptomatic pulmonary embolism

**DOI:** 10.1080/07853890.2025.2529570

**Published:** 2025-07-09

**Authors:** Wei Xiong, Qiangqiang Qin, Xiaoyang Song, Zhenzhong Deng, Mei Xu, Dongmei Wang, Qihuan Yao, Jianmin Qu, Yong Luo, Fengfeng Han

**Affiliations:** a Department of Pulmonary and Critical Care Medicine, Xinhua Hospital, Shanghai Jiaotong University School of Medicine, Shanghai, China; b Department of Cardiovascular Medicine, Graduate School of Medicine, Kyoto University, Kyoto, Japan; c Department of Pulmonary and Critical Care Medicine, Chongming Hospital, Shanghai University of Medicine and Health Sciences, Shanghai, China; d Department of Oncology, Xinhua Hospital, Shanghai Jiaotong University School of Medicine, Shanghai, China; e Department of General Practice, North Bund Community Health Service Center, Hongkou District, Shanghai, China; f Department of Traditional Chinese Medicine, Kongjiang Hospital, Yangpu District, Shanghai, China; g Department of Critical Care Medicine, Tongxiang First People’s Hospital, Tongxiang, China

**Keywords:** Pulmonary embolism, severity, outcomes, symptom, onset to diagnosis, identification

## Abstract

**Background:**

The more severe the pulmonary embolism (PE), the shorter the time interval from PE symptom onset to diagnosis (OTD). Nevertheless, it is not known how many days of OTD is the optimal threshold for the identification of severe PE or what differences exist in outcomes among PE patients classified by this threshold.

**Methods:**

Patients with acute symptomatic PE were retrospectively studied to determine the optimal OTD threshold for identifying the severity and outcomes of PE. The differences in one-year mortality, VTE recurrence, major bleeding, and composite outcomes among patients with PE classified by this threshold were compared.

**Results:**

A total of 1878 patients with PE were finally obtained. All patients were divided into the short OTD (OTD ≤ 1day) group (*N* = 736) and long OTD (OTD > 1day) group (*N* = 1142), based on the acquired OTD threshold of one day. The short OTD group had more shock (20.7% vs. 2.9%), hypoxia (62.8% vs. 34.0%), and cardiac arrest (9.2% vs. 1.2%) at PE diagnosis than the long OTD group (all *p* < 0.001). The occurrence of one-year all-cause mortality (21.7% vs. 16.5%, *p* = 0.004), PE-related mortality (7.9% vs. 1.9%, *p* < 0.001), and composite outcomes (28.7% vs. 23.0%, *p* = 0.006) in the short OTD group were more than that in the long OTD group. In multivariable analyses, OTD > 1day was correlated with a decreased risk of high-risk PE (OR 0.263 [0.117–0.591], *p* = 0.001) and one-year composite outcomes (HR 0.812 [0.677–0.974], *p* = 0.025), compared with OTD ≤ 1day.

**Conclusions:**

PE patients with OTD ≤ 1day had more high-risk PE and worse one-year clinical outcomes, compared to those with OTD > 1day. An OTD of one day could be the optimal threshold for the identification of severity and outcomes of PE.

## Introduction

Acute pulmonary embolism (PE) has a high morbidity and mortality rate, which poses a threat to the life and health of people worldwide [1–3]. Understanding the risk factors associated with the severity and outcomes of patients with PE is helpful for their management. Previous literature reported that PE patients with hypotension and dyspnea were prone to present to doctors earlier [4]. PE patients in the absence of syncope or sudden dyspnea were prone to form delayed PE diagnosis [5]. The presence of more severe clinical presentations was associated with a more timely objective diagnosis [6]. In other words, the more severe the PE, the shorter the time interval from PE symptom onset to diagnosis (OTD). Nevertheless, it was unclear how many days of OTD is the optimal threshold for identifying whether PE is severe or not, or what differences exist in clinical outcomes among PE patients categorized based on this threshold. Several previous studies have defined one week of OTD as the threshold to distinguish delayed PE diagnosis from undelayed PE diagnosis, and reported that there was no difference in mortality or VTE recurrence between patients classified according to this threshold [7,8]. We were uncertain whether a 7-day OTD was the optimal threshold for the identification of PE severity at diagnosis and prognostic outcomes of acute symptomatic PE, and hypothesized that there may be an optimal threshold. Therefore, this study was conducted to address these issues.

## Methods

### Study protocol

Patients with International Classification of Diseases 10th revision (ICD-10) code [9] (I26) were retrospectively reviewed. OTD was defined as the time interval from the initial onset of PE symptoms to the final objective imaging diagnosis. Eligible patients were hospitalized adults who had a discharge diagnosis of objectively confirmed acute symptomatic PE^3^ and completed standard treatment, defined as a minimum of 3 months of anticoagulation [3], and were followed up for at least one year after PE diagnosis. The correlation between the OTD of all eligible patients and severe PE which was defined as high-risk PE was analyzed. High-risk PE was defined as PE patients with hemodynamic instability which comprised cardiac arrest, obstructive shock, and persistent hypotension, according to the 2019 European Society of Cardiology (ESC) PE guidelines [3]. In the correlation analysis between OTD and high-risk PE as well as cardiac arrest at diagnosis, an OTD threshold was acquired to divide the patients into short and long OTD groups. The different OTD groups corresponded to severe (high-risk) and non-severe (non-high-risk) PE. Clinical outcomes including all-cause death, PE-related death [10], VTE recurrence [11], major bleeding [12], and composite outcomes 3 months and 1 year after PE diagnosis were compared between the short and long OTD groups. Composite outcomes were defined as the composite of death, VTE recurrence, and major bleeding. Correlations between OTD and clinical outcomes 3 months and 1 year after PE diagnosis were also analyzed. The role of the acquired OTD threshold was compared to that of a 7-day OTD threshold [7,8].

### Study population

The eligible study population was selected based on the following inclusion and exclusion criteria. The inclusion criteria were as follows: (1) patients were older than 18 years at the time of PE diagnosis. (2) Patients were discharged with an objective diagnosis of acute symptomatic PE through computed tomography pulmonary angiography (CTPA) or planar ventilation/perfusion (V/Q) scan [3]. (3) Patients completed standard treatment, which was defined as a minimum of 3 months of anticoagulation for all patients and a minimum of 6 months of anticoagulation for patients with active cancer [3], and were followed up for at least one year after PE diagnosis. Extended anticoagulation beyond the standard treatment course was performed at the discretion of attending physicians of the patients. The exclusion criteria were as follows: (1) patients had a definite history of chronic thromboembolic disease (CTED) or chronic thromboembolic pulmonary hypertension (CTEPH) [3]. (2) Patients were terminally ill, defined as having a life expectancy of less than 6 months [13] at the time of PE diagnosis.

### Study ethics

The study protocol was approved by the Ethics Committee of the Shanghai Xinhua Hospital (approval number: XHEC-QT-2024-059). Written informed consent from the participants or their next of kin was waived because: (1) the study involved no more than minimal risk for patients; (2) the study cannot adversely affect the rights and welfare of patients; and (3) the study cannot be performed without the exemption of informed consent of patients. All the authors contributed to this work and approved the submitted version of the manuscript. None of the authors have contributed to the manuscript. The overall reporting of the current study was in line with the Strengthening the Reporting of Observational Studies in Epidemiology (STROBE) statement [14].

### Statistical analysis

Continuous variables were presented as mean and standard deviation or as median and interquartile range (IQR), based on whether their distributions were normal. Shapiro–Wilk test was used in the normality test. Categorical variables are presented as numbers and percentages. Continuous variables were compared using Student *t*-test or Mann–Whitney *U* test based on whether their distributions were normal, whereas the comparison of categorical variables was conducted using χ^2^ test or Fisher exact test. Missing data were addressed by using multiple imputations.

Receiver operating characteristic (ROC) analysis was used to obtain and validate the threshold of OTD for the identification of high-risk PE. Sequential univariable and multivariable binary Logistic regression analysis were used to explore the odds ratios between OTD and high-risk PE at diagnosis, incorporating the risk factors for high-risk PE indicated by the ESC guidelines [3] as covariates, which included age (>80 y vs. ≤80 y), sex (women vs. men), active cancer (yes vs. no), chronic heart failure (yes vs. no), chronic pulmonary diseases (yes vs. no), heart rate (≥110bpm vs.<110bpm), hypoxia (yes vs. no), right ventricular dysfunction (yes vs. no), and troponin I (≥45 pg/mL vs.<45 pg/mL). Sequential univariable and multivariable Cox regression analysis was used to explore the hazard ratios between OTD and 1-year composite outcomes which was defined as the composite of death, VTE recurrence, and major bleeding. Since there are no suggested risk factors for long-term composite outcomes of PE in guidelines, the covariates we chose comprised demographics variables including age (>80 y vs.≤80y) and sex (women vs. men), comorbidities including active cancer (yes vs. no), chronic heart failure (yes vs. no), chronic pulmonary diseases (yes vs. no), history of stroke (yes vs. no), and hypertension (yes vs. no), recurrence-related factor like history of VTE (yes vs. no), and bleeding-related factor like history of major bleeding (yes vs. no).

PE severity including shock, tachycardia, hypoxia, cardiac arrest, and simplified Pulmonary Embolism Severity Index (sPESI) ≥1 at PE diagnosis were compared between the short and long OTD groups, using χ^2^ test. Kaplan–Meier curve analysis was used to compare the cumulative time-dependent clinical outcomes including all-cause death, PE-related death, VTE recurrence, major bleeding, and composite outcomes 3 months and 1 year after PE diagnosis, between the short and long OTD groups. All statistical analyses were conducted using SPSS 27 software. Statistical significance was defined as a two-tailed *P*-value of < 0.05.

## Results

### Patient characteristics

**Figure 1.**
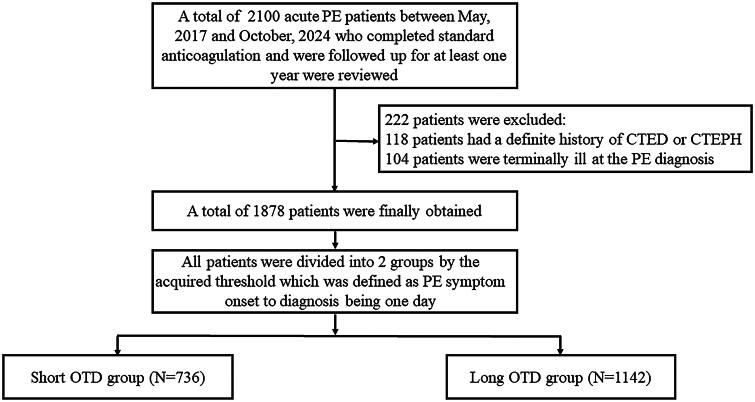
Study flowchart. PE: pulmonary embolism; CTED: chronic thromboembolic disease; CTEPH: chronic thromboembolic pulmonary hypertension; OTD: onset to diagnosis.

A total of 2100 hospitalized patients with acute symptomatic PE between May 2017 and October 2024 who completed standard anticoagulation therapy and were followed up for at least one year were reviewed, following the inclusion criteria. A total of 1878 patients were finalized after the exclusion of 118 patients who had a definite history of CTED or CTEPH, and 104 patients who were terminally ill at PE diagnosis, following the exclusion criteria. In the correlation analysis between OTD and high-risk PE as well as cardiac arrest at PE diagnosis, an OTD threshold of one day was obtained. All patients were divided into the short OTD group (*N* = 736) which was defined as an OTD ≤ 1day, and the long OTD group (*N* = 1142) which was defined as an OTD > 1day, according to the acquired OTD threshold. The flowchart of the study is shown in Figure 1.

The frequency of the occurrence of different OTDs in all patients is shown in Figure S1. Patients who had shock, hypoxia, and cardiac arrest at PE diagnosis or died within one year after PE diagnosis had a shorter OTD than those who did not (Table S1). Among patients with 1–7 days of OTD, those with 1-day OTD had the highest proportion of shock, hypoxia, cardiac arrest, and PE-related mortality (Figure S2). The median OTD in all patients was 3.0 (IQR [1.0–7.0]) days. The OTD in the short and long OTD groups were 0.0 (IQR [0.0-1.0]) days and 7.0 (IQR [3.0–12.0]) days, respectively (*p* < 0.001). Compared with the long OTD group, the short OTD group included older patients and more female. There was no statistical difference in the medical history or treatment between the two groups. Patient characteristics of the short and long OTD groups are shown in Table 1.

**Table 1.** Characteristics between short and long OTD groups.

	Short OTD group (*N* = 736)	Long OTD group (*N* = 1142)	*P*-value
Demographics			
Age – years	70 (60–78)	68 (57–77)	0.011
Male – no. (%)	259 (35.2)	470 (41.2)	0.010
Body mass index – kg/m^2^	22.8 (20.2–25.6)	23.0 (20.5–25.5)	0.393
Medical history – no. (%)			
Active cancer	237 (32.2)	334 (29.2)	0.174
VTE history	38 (5.2)	58 (5.1)	0.936
Chronic heart failure	18 (2.4)	36 (3.2)	0.371
Chronic pulmonary diseases	82 (11.1)	129 (11.3)	0.917
Stroke history	86 (11.7)	84 (7.4)	0.001
Active autoimmune diseases	32 (4.3)	88 (7.7)	0.004
Thrombophilia	30 (4.1)	85 (7.4)	0.003
Varicose veins	20 (2.7)	43 (3.8)	0.218
Bleeding diathesis	82 (11.1)	75 (6.6)	<0.001
Anticoagulation before PE	33 (4.5)	58 (5.1)	0.558
Vital signs – no. (%)			
Shock	152 (20.7)	33 (2.9)	<0.001
Tachycardia	142 (19.3)	121 (10.6)	<0.001
Hypoxia	462 (62.8)	388 (34.0)	<0.001
Cardiac arrest	68 (9.2)	14 (1.2)	<0.001
PE characteristics			
Onset to diagnosis – days	0.0 (0.0–1.0)	7.0 (3.0–12.0)	<0.001
Out-of-hospital onset – no. (%)	462 (62.8)	974 (85.3)	<0.001
PE with DVT – no. (%)	493 (67.0)	932 (81.6)	<0.001
Provoked PE – no. (%)	323 (43.9)	298 (26.1)	<0.001
High-risk PE – no. (%)	152 (20.7)	33 (2.9)	<0.001
sPESI ≥1 – no. (%)	657 (89.3)	832 (72.9)	<0.001
sPESI – points	2.0 (1.0–2.0)	1.0 (0.0–2.0)	<0.001
Laboratory tests			
D-dimer – mg/L	1.4 (0.7–3.0)	1.1 (0.6–2.0)	<0.001
Platelet – 10^9^/L	189.0 (147.0–241.3)	197.0 (156.0–250.8)	0.380
Hemoglobin – g/L	118.1 ± 23.6	124.0 ± 23.4	<0.001
Creatine – μmol/L	74.0 (60.0–100.0)	74.0 (60.0–94.0)	0.518
NT-proBNP – pg/mL	1145.0 (353.0–3187.0)	1085.0 (338.0–3572.0)	0.720
TNI – ng/mL	0.08 (0.03–0.47)	0.05 (0.01–0.15)	0.011
Treatment – no. (%)			
Rivaroxaban	564 (76.6)	878 (76.9)	0.899
Edoxaban	165 (22.4)	248 (21.7)	0.720
Other anticoagulants	7 (1.0)	16 (1.4)	0.387
Thrombolysis	86 (11.7)	23 (2.0)	<0.001
Concomitant antiplatelets	60 (8.2)	94 (8.2)	0.951
Extended OA beyond 3 months	276 (37.5)	471 (47.2)	0.106

According to the European Society of Cardiology (ESC) guidelines for PE^3^, shock was defined as a systolic blood pressure (BP) < 90 mmHg or vasopressors required to achieve a BP ≥ 90 mmHg, or systolic BP drop ≥ 40mmHg lasting longer than 15 min and not caused by new-onset arrhythmia, hypovolaemia, or sepsis. Tachycardia was defined as a heart rate ≥110 times/min. Hypoxia was defined as a peripheral oxygen saturation <90% or an arterial partial pressure of oxygen <60 mmHg. OTD: onset to diagnosis; VTE: venous thromboembolism; PE: pulmonary embolism; DVT: deep vein thrombosis; sPESI: simplified pulmonary embolism severity index; NT-proBNP: N-terminal pro B-type natriuretic peptide; TNI: troponin I; OA: oral anticoagulant.

### Correlation between OTD and PE severity as well as outcomes

**Figure 2.**
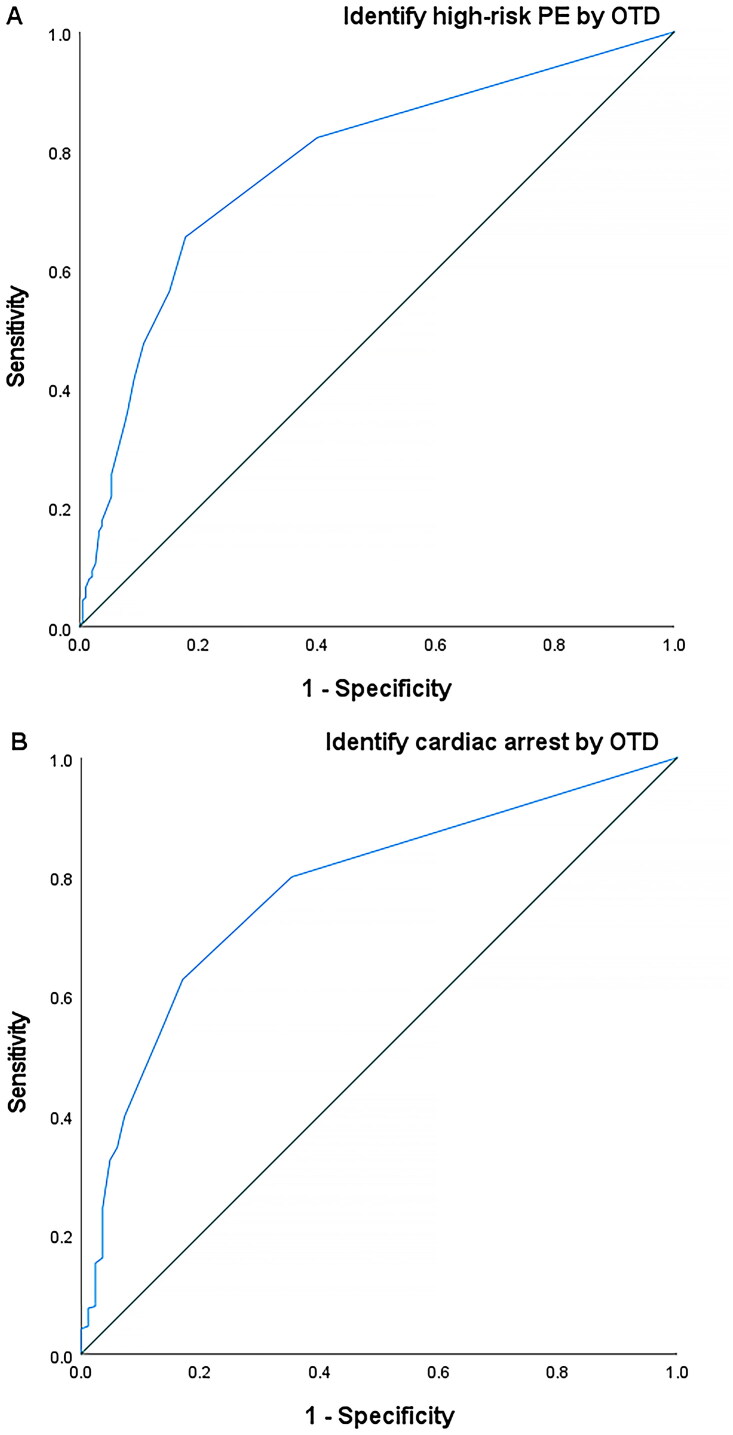
ROC curve for identification of high-risk PE (A) and cardiac arrest (B) at PE diagnosis using OTD. ROC: receiver operating characteristic; PE: pulmonary embolism; OTD: onset-to-diagnosis.

The ROC curve analysis demonstrated that the area under the curve (AUC), sensitivity, specificity, and cutoff values were 0.776 (95% confidence interval [CI] [0.741–0.810]),65.5%, 82.2%, and 1 day, respectively, when the incremental continuous values of OTD were used to identify the absence of high-risk PE at PE diagnosis (*p* < 0.001) (Figure 2A). The ROC curve analysis demonstrated that the AUC, sensitivity, specificity, and cutoff value were 0.778 (95% CI [0.731–0.825]), 62.8%, 82.9%, and 1 day, respectively, when incremental continuous values of OTD were used to identify the absence of cardiac arrest at PE diagnosis (*p* < 0.001) (Figure 2B).

In the univariable Logistic regression analysis, OTD > 1day was correlated with a decreased risk of high-risk PE compared with OTD ≤ 1day (OR 0.114 [0.077–0.169], *p* < 0.001). Such a correlation remained similar in the multivariable Logistic regression analysis (OR 0.263 [0.117–0.591], *p* = 0.001) (Table S2). In the univariable Cox regression analysis, OTD > 1day was correlated with a decreased risk of one-year composite outcomes compared with OTD ≤ 1day (HR 0.728 [0.608-0.873], *p* = 0.001). Such a correlation remained similar in the multivariable Cox regression analysis (HR 0.812 [0.677-0.974], *p* = 0.025) (Table S3).

### Comparison of PE severity and outcomes between short and long OTD groups

There were 152 (20.7%) and 33 (2.9%) patients with high-risk PE or shock at PE diagnosis in the short and long OTD groups, respectively (*p* < 0.001). There were 142 (19.3%) and 121 (10.6%) patients with tachycardia at PE diagnosis in the short and long OTD groups, respectively (*p* < 0.001). There were 462 (62.8%) and 388 (34.0%) patients with hypoxia at PE diagnosis in the short- and long-OTD groups, respectively (*p* < 0.001). There were 68 (9.2%) and 14 (1.2%) patients with cardiac arrest at PE diagnosis in the short and long OTD groups, respectively (*p* < 0.001). There were 657 (89.3%) and 832 (72.9%) patients with sPESI ≥ 1 at PE diagnosis in the short and long OTD groups, respectively (*p* < 0.001). A comparison of PE severity between the short and long OTD groups is presented in Table 1.

**Figure 3.**
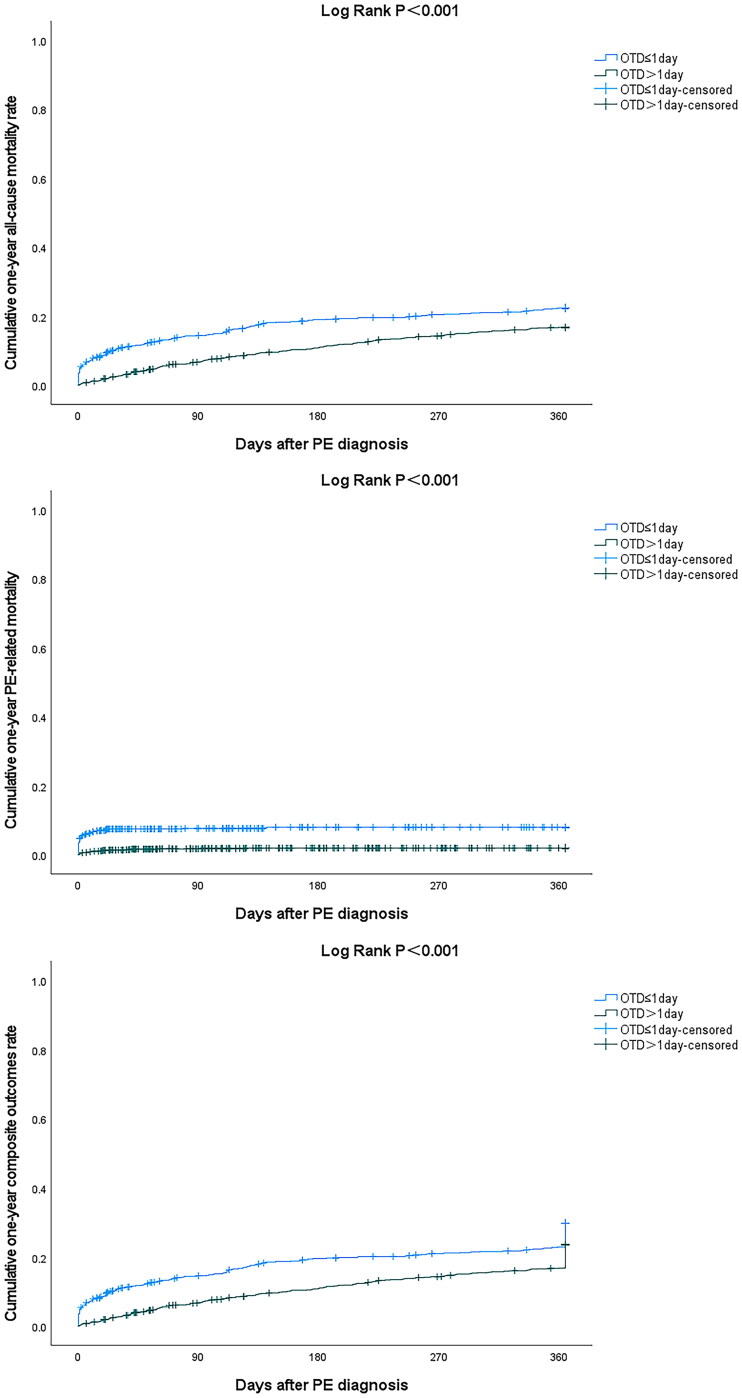
Kaplan–Meier curve comparing one-year all-cause mortality, PE-related mortality and composite outcomes between short and long OTD groups. Composite outcomes were defined as the composite of death, VTE recurrence, and major bleeding. PE: pulmonary embolism; OTD: onset to diagnosis.

As of the time of the current study, the median follow-up period for all the patients was 26.1 (IQR [6.2–46.9]) months, among which the follow-up period of the short OTD group was 18.3 (IQR [2.4–40.4]) months, whereas that of the long OTD group was 29.9 (IQR [9.4–50.4]) months. The occurrence of 3-month all-cause mortality (105 [14.3%] vs. 77 [6.7%], *p* < 0.001), PE-related mortality (56 [7.6%] vs. 20 [1.8%], *p* < 0.001), and composite outcomes (145 [19.7%] vs. 134 [11.7%], *p* < 0.001) were higher in the short OTD group than in the long OTD group. The occurrence of 1-year all-cause mortality (160 [21.7%] vs. 188 [16.5%], *p* = 0.004), PE-related mortality (58 [7.9%] vs. 22 [1.9%], *p* < 0.001), and composite outcomes (211 [28.7%] vs. 263 [23.0%], *p* = 0.006) was higher in the short OTD group than in the long OTD group. There was no statistical difference in the comparison of other variables between the two groups. A comparison of the outcomes between the short and long OTD groups is presented in Table 2. The Kaplan–Meier curve analysis demonstrated that the one-year cumulative time-dependent incidence of all-cause mortality (HR 0.689, 95%CI [0.558–0.851], log-rank *p* < 0.001), PE-related mortality (HR 0.234, 95%CI [0.143–0.382], log-rank *p* < 0.001), and composite outcomes (HR 0.728, 95%CI [0.608–0.873], log-rank *p* < 0.001) in the long OTD group were lower than those in the short OTD group (Figure 3).

**Table 2.** Outcomes at 3-month and 1-year after PE diagnosis between short and long OTD groups.

	Short OTD group (*N* = 736)	Long OTD group (*N* = 1142)	*P*-value
3-month outcomes – no. (%)			
All-cause mortality	105 (14.3)	77 (6.7)	<0.001
PE-related mortality	56 (7.6)	20 (1.8)	<0.001
VTE recurrence	23 (3.1)	37 (3.2)	0.890
Major bleeding	42 (5.7)	44 (3.9)	0.061
Composite outcomes	145 (19.7)	134 (11.7)	<0.001
1-year outcomes – no. (%)			
All-cause mortality	160 (21.7)	188 (16.5)	0.004
PE-related mortality	58 (7.9)	22 (1.9)	<0.001
VTE recurrence	34 (4.6)	70 (6.1)	0.162
Major bleeding	52 (7.1)	76 (6.7)	0.731
Composite outcomes	211 (28.7)	263 (23.0)	0.006

Composite outcomes were defined as a composite of death, VTE recurrence, and major bleeding. PE: pulmonary embolism; OTD: onset to diagnosis; VTE: venous thromboembolism.

### One-day OTD vs seven-day OTD

The ROC analysis demonstrated that the AUC were 0.738 (95%CI [0.703–0.774], *p* < 0.001) and 0.729 (95%CI [0.678–0.779], *p* < 0.001) when the OTD threshold of 1 day was used to identify the absence of high-risk PE and cardiac arrest at PE diagnosis, respectively. In contrast, the ROC analysis demonstrated that the AUC was 0.601 (95%CI [0.564–0.639], *p* < 0.001) and 0.604 (95%CI [0.552–0.657], *p* = 0.001) when the threshold OTD of 7 days was used to identify the absence of high-risk PE and cardiac arrest at PE diagnosis, respectively.

When patients were grouped based on a 7-day OTD threshold, although there were still differences in PE severity between the two groups (Table S4), the proportion of high-risk PE or shock, hypoxia, and cardiac arrest in the short OTD group was lower than that in the short OTD group by the 1-day OTD threshold. When patients were divided by a 7-day OTD threshold, only PE-related mortality occurred more in the short OTD group than in the long OTD group in terms of one-year clinical outcomes (Table S4). The Kaplan-Meier curve comparing the one-year PE-related mortality rates between patients classified by a 7-day OTD threshold is presented in Figure S3.

## Discussion

The major findings in the current study included the following: (1) An OTD of one day was the threshold for identifying whether patients had high-risk PE or not. (2) Patients with an OTD ≤ 1 day had more high-risk PE at PE diagnosis than those with an OTD > 1 day. PE patients with an OTD ≤ 1 day had higher rates of composite clinical outcomes, than those with an OTD > 1 day. OTD was an independent risk factor of the severity and outcomes of PE. (3) An OTD of one day was more suitable than an OTD of seven days for identifying PE severity at PE diagnosis and prognostic clinical outcomes.

To the best of our knowledge, this is the first study on this topic, as no comparable previous studies have been conducted. The current study reproduced findings that were basically consistent with those in previous reports [4–6], in which the time interval from PE symptom onset to diagnosis was negatively correlated with PE severity in patients with acute symptomatic PE. In the ROC curve analysis, an OTD ≤ 1day indicated a high likelihood of high-risk PE or cardiac arrest, whereas an OTD > 1day indicated a low likelihood of high-risk PE or cardiac arrest. The specificity was higher than the sensitivity, suggesting that the OTD threshold of one day is more suitable for identifying the likelihood of high-risk PE or cardiac arrest, namely, a high-risk PE or cardiac arrest is likely when the OTD is one day or less. OTD can be affected by two categories of factors: one is patient-related factors, the other is hospital-related factors. Patient-related factors refer to the timeliness of patients seeking medical attention after PE onset, whereas hospital-related factors refer to the timeliness of hospitals in clarifying PE diagnosis after patients seek medical attention [15]. It is reasonable that PE patients with severe symptoms will seek medical attention sooner than those without. By the same token, clinicians would usually prioritize patients with suspected PE and severe symptoms and expedite the diagnostic process for them, in contrast to those without severe symptoms.

During the follow-up, patients diagnosed with PE within one day after symptom onset had higher long-term mortality rates, instead of VTE recurrence and major bleeding, than those diagnosed more than one day after symptom onset. This is consistent with the findings with respect to VTE recurrence in previous studies [7,8] in which the OTD threshold was 7 days, whereas it was inconsistent with their findings in terms of mortality. This could be because an OTD of 1 day outperformed an OTD of 7 days for identifying PE severity at diagnosis and prognostic outcomes. Previous studies reported that PE patients at a high-risk stratification of sPESI or PESI score which represents severe PE had worse one- or five-year mortality, compared with those at a low-risk stratification of sPESI or PESI score [16,17], suggesting that the more severe the PE at diagnosis, the worse the long-term mortality. The current findings are consistent with those in previous literature [16,17]. Severe PE can shorten OTD and lead to adverse outcomes. Rapidly diagnosed acute symptomatic PE is a sign of severe PE and poor outcomes for the patients. However, this merely reflects the causal relationship from the severity of disease to the timeliness of diagnosis, instead of reflecting the causal relationship from the timeliness of diagnosis to the severity of disease. It is not the early diagnosis that makes the condition of patients severe, or the late diagnosis that makes the condition of patients mild. Prolonging OTD cannot improve the severity or outcomes of PE, whereas shortening OTD cannot worsen the severity or outcomes of PE.

The current findings have several clinical implications. First, due to the fact that a one-day OTD remains an independent risk factor for the severity and outcomes of PE patients, after excluding the interference of many other confounding factors, it could be considered as a new clinical hallmark for PE which may have certain role in the assessment of PE severity and outcomes. Clinicians may need to be vigilant that a rapidly diagnosed PE within one day after symptom onset may indicate severe PE and poor prognosis. Second, compared with PE patients with other OTDs, there was the highest proportion of severe PE and mortality among patients with an OTD of one day. An OTD of one day may be considered as the optimal threshold for distinguishing delayed PE diagnosis from undelayed PE diagnosis, because the PE diagnosis more than one day after symptom onset may delay the treatment of most severe PE patients. This is also consistent with a previous study, in which a delayed PE diagnosis was defined as the diagnosis being confirmed more than 24 h after the PE symptoms onset [15]. Regardless of the situation, PE patients should be diagnosed as soon as possible after symptom onset. If a threshold must be defined, it is optimal for patients to receive a definite PE diagnosis within 24 h after the onset of PE symptoms.

### Limitations

This study has some limitations that need to be acknowledged. First, this was a retrospective, observational study. The retrospective nature of this study is a limitation. Second, because the time of initial PE symptom onset in patients was based on their chief complaints in inpatient medical records, whether its accuracy was absolutely reliable cannot be completely certain. Third, this study did not divide the OTD of PE patients into two phases: the phase from symptom onset to first seeking medical attention and the phase from first seeking medical attention to diagnosis because the time of patients’ first seeking medical attention was difficult to accurately determine. In this context, it is unknown which of the patient-related and hospital-related factors has a greater impact on the OTD. Nevertheless, this had little impact on the credibility of the current research findings. Fourth, because all PE patients in this study were hospitalized, caution should be exercised when extrapolating the current findings to PE patients receiving outpatient treatment. Lastly, since all the PE patients in this study were Chinese from medical institutions in China, extra caution is needed when extrapolating the research findings to other countries because medical policies, medical level, and the principles of diagnosis and treatment may vary from country to country.

## Conclusions

Rapidly diagnosed PE could be a sign of severe PE and poor outcomes for the patients. OTD is an independent risk factor of the PE severity at diagnosis and of one-year prognostic clinical outcomes. PE patients with OTD ≤ 1day had higher PE severity at diagnosis and higher one-year mortality rate, compared to those with OTD > 1day. An OTD of one day could be the optimal threshold for the identification of PE severity at diagnosis and of long-term clinical outcomes, and even for the discrimination of delayed PE diagnosis from an undelayed one.

## Supplementary Material

Supplemental Material

## Data Availability

Data are available from the corresponding authors upon reasonable request.
