# Supplementary material for: Optimal threshold of time interval from symptom onset to diagnosis for identification of severity and outcomes in acute symptomatic pulmonary embolism
Source: Ann Med. 2025 Jul 9;57(1):2529570. doi: 10.1080/07853890.2025.2529570 (PMC12247100; doi:10.1080/07853890.2025.2529570)
Supplement: Supplemental Material [file IANN_A_2529570_SM7371.docx]

**Supplementary Material**

**Supplementary tables**

| **Table S1-Comparison of OTD between different PE severity and between different one-year outcomes** | | | |
| --- | --- | --- | --- |
|  | **OTD in positive patients (days)** | **OTD in negative patients (days)** | **P value** |
| **PE severity at diagnosis** |  |  |  |
| High-risk PE or shock | 0.0 (0.0-1.0) | 3.0 (1.0-8.0) | ＜0.001 |
| Hypoxia | 1.0 (0.0-5.0) | 4.0 (1.0-10.0) | ＜0.001 |
| Cardiac arrest | 0.0 (0.0-1.0) | 3.0 (1.0-7.0) | ＜0.001 |
| **One-year outcomes** |  |  |  |
| All-cause mortality | 2.0 (0.0-7.0) | 3.0 (1.0-7.0) | 0.024 |
| PE-related mortality | 0.0 (0.0-3.0) | 3.0 (1.0-7.0) | ＜0.001 |
| VTE recurrence | 4.0 (1.0-8.0) | 3.0 (1.0-7.0) | 0.109 |
| Major bleeding | 3.0 (1.0-8.5) | 3.0 (1.0-7.0) | 0.964 |
| Composite outcomes | 2.0 (1.0-7.0) | 3.0 (1.0-7.0) | 0.072 |

Abbreviations: OTD: onset to diagnosis；PE：pulmonary embolism; VTE: venous thromboembolism

| **Table S2-** **Correlation between OTD and high-risk PE at diagnosis** | | | | |
| --- | --- | --- | --- | --- |
|  | **Univariable**  **OR (95%CI)** | **P value** | **Multivariable**  **OR (95%CI)** | **P value** |
| **Age (＞80y vs.≤80y)** | 1.597 (1.105-2.308) | 0.013 | 1.109 (0.467-2.633) | 0.815 |
| **Sex (women vs. men)** | 1.325 (0.960-1.827) | 0.087 |  |  |
| **Active cancer (yes vs. no)** | 0.448 (0.302-0.664) | ＜0.001 | 0.888 (0.314-2.510) | 0.823 |
| **Chronic heart failure (yes vs.**  **no)** | 0.531 (0.164-1.717) | 0.290 |  |  |
| **Chronic pulmonary diseases (yes vs. no)** | 0.472 (0.252-0.883) | 0.019 | 0.124 (0.015-0.994) | 0.049 |
| **Heart rate (≥110bpm vs.＜110bpm)** | 4.387 (3.060-6.290) | ＜0.001 | 1.710 (0.694-4.210) | 0.243 |
| **Hypoxia (yes vs. no)** | 33.53 (16.40-68.55) | ＜0.001 | 14.12 (3.102-64.26) | 0.001 |
| **Right ventricular dysfunction (yes vs. no)** | 3.294 (2.379-4.561) | ＜0.001 | 1.620 (0.643-4.083) | 0.306 |
| **Troponin I (**≥**45 pg/mL vs.＜45 pg/mL)** | 2.177 (1.101-4.301) | 0.025 | 1.127 (0.480-2.646) | 0.783 |
| **OTD(＞1day vs.≤1day)** | 0.114 (0.077-0.169) | ＜0.001 | 0.263 (0.117-0.591) | 0.001 |

Abbreviations: OTD: onset to diagnosis；OR: odds ratio; CI: confidence interval; y: years; bpm: beat per minute

| **Table S3-** **Correlation between OTD and** **one-year composite outcomes** | | | | |
| --- | --- | --- | --- | --- |
|  | **Univariable**  **HR (95%CI)** | **P value** | **Multivariable**  **HR (95%CI)** | **P value** |
| **Age (＞80y vs.≤80y)** | 1.509 (1.206-1.888) | ＜0.001 | 2.188 (1.730-2.767) | ＜0.001 |
| **Sex (women vs. men)** | 0.900 (0.750-1.081) | 0.260 |  |  |
| **Active cancer (yes vs. no)** | 4.134 (3.439-4.968) | ＜0.001 | 4.650 (3.841-5.629) | ＜0.001 |
| **Chronic heart failure (yes vs.**  **no)** | 0.856 (0.483-1.518) | 0.595 |  |  |
| **Chronic pulmonary diseases (yes vs. no)** | 1.298 (0.999-1.686) | 0.051 |  |  |
| **History of stroke (yes vs. no)** | 1.358 (1.021-1.808) | 0.036 | 1.554 (1.112-2.173) | 0.010 |
| **Hypertension (yes vs. no)** | 0.814 (0.676-0.982) | 0.031 | 0.771 (0.635-0.935) | 0.008 |
| **History of VTE** **(yes vs. no)** | 1.247 (0.856-1.815) | 0.250 |  |  |
| **History of major bleeding (yes vs. no)** | 1.652 (1.255-2.175) | ＜0.001 | 1.270 (0.929-1.735) | 0.134 |
| **OTD(＞1day vs.≤1day)** | 0.728 (0.608-0.873) | 0.001 | 0.812 (0.677-0.974) | 0.025 |

Abbreviations: OTD: onset to diagnosis; HR: hazard ratio; CI: confidence interval; y: years; VTE: venous thromboembolism

| **Table S4- PE severity and Outcomes between short and long OTD groups by a 7-day OTD** | | | |
| --- | --- | --- | --- |
|  | **Short OTD group**  **(N=1434)** | **Long OTD group**  **(N=444)** | **P value** |
| **PE severity - no. (%)** |  |  |  |
| High-risk PE or shock | 175 (12.2) | 10 (2.3) | <0.001 |
| Hypoxia | 717 (50.0) | 133 (30.0) | <0.001 |
| Cardiac arrest | 79 (5.5) | 3 (0.7) | <0.001 |
| **3-month outcomes - no. (%)** |  |  |  |
| All-cause mortality | 154 (10.7) | 28 (6.3) | 0.006 |
| PE-related mortality | 70 (4.9) | 6 (1.4) | <0.001 |
| VTE recurrence | 44 (3.1) | 16 (3.6) | 0.575 |
| Major bleeding | 67 (4.7) | 19 (4.3) | 0.729 |
| Composite outcomes | 145 (19.7) | 134 (11.7) | 0.094 |
| **1-year outcomes - no. (%)** |  |  |  |
| All-cause mortality | 277 (19.3) | 71 (16.0) | 0.115 |
| PE-related mortality | 74 (5.2) | 6 (1.4) | <0.001 |
| VTE recurrence | 77 (5.4) | 27 (6.1) | 0.567 |
| Major bleeding | 93 (6.5) | 35 (7.9) | 0.307 |
| Composite outcomes | 366 (25.5) | 108 (24.3) | 0.611 |

Abbreviations: PE: pulmonary embolism; OTD: onset to diagnosis; VTE: venous thromboembolism

**Figure S1**- Frequency of occurrence of different OTDs in all patients Abbreviations: PE: pulmonary embolism; OTD: onset to diagnosis


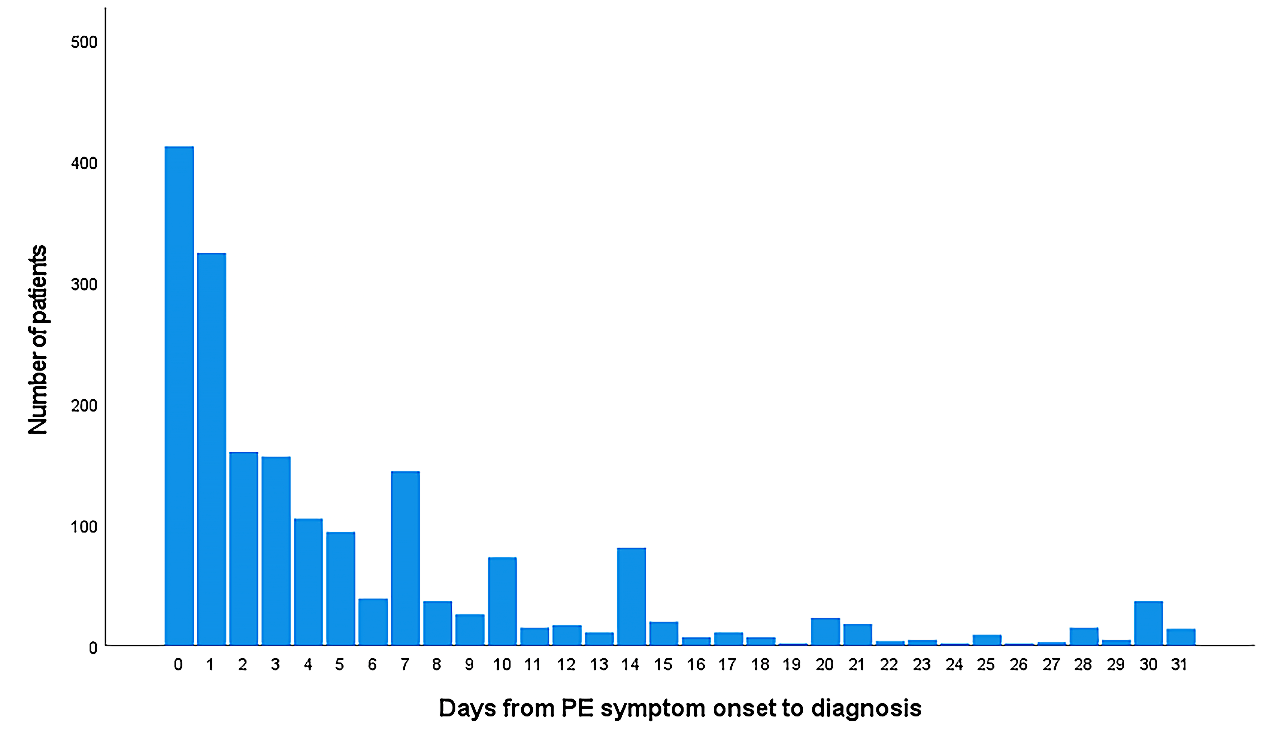


**Figure S2**-Proportion of PE severity and outcomes among patients with 1 to 7 days of OTD

Abbreviations: PE: pulmonary embolism; VTE: venous thromboembolism; OTD: onset to diagnosis


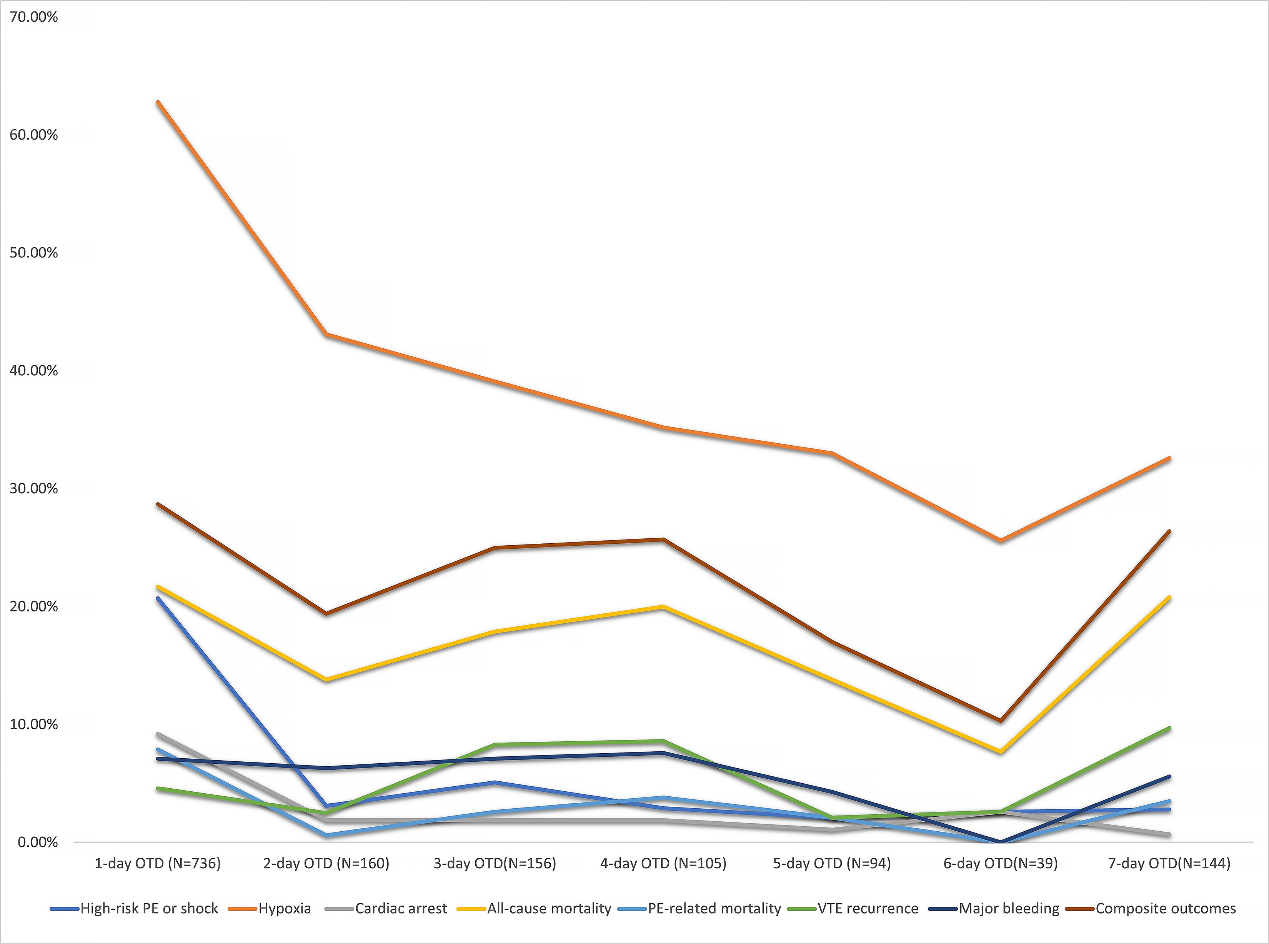


**Figure S3**- Kaplan-Meier curve comparing 1-year PE-related mortality between patients classified by a 7-day OTD

Abbreviations: PE: pulmonary embolism; OTD: onset to diagnosis

**
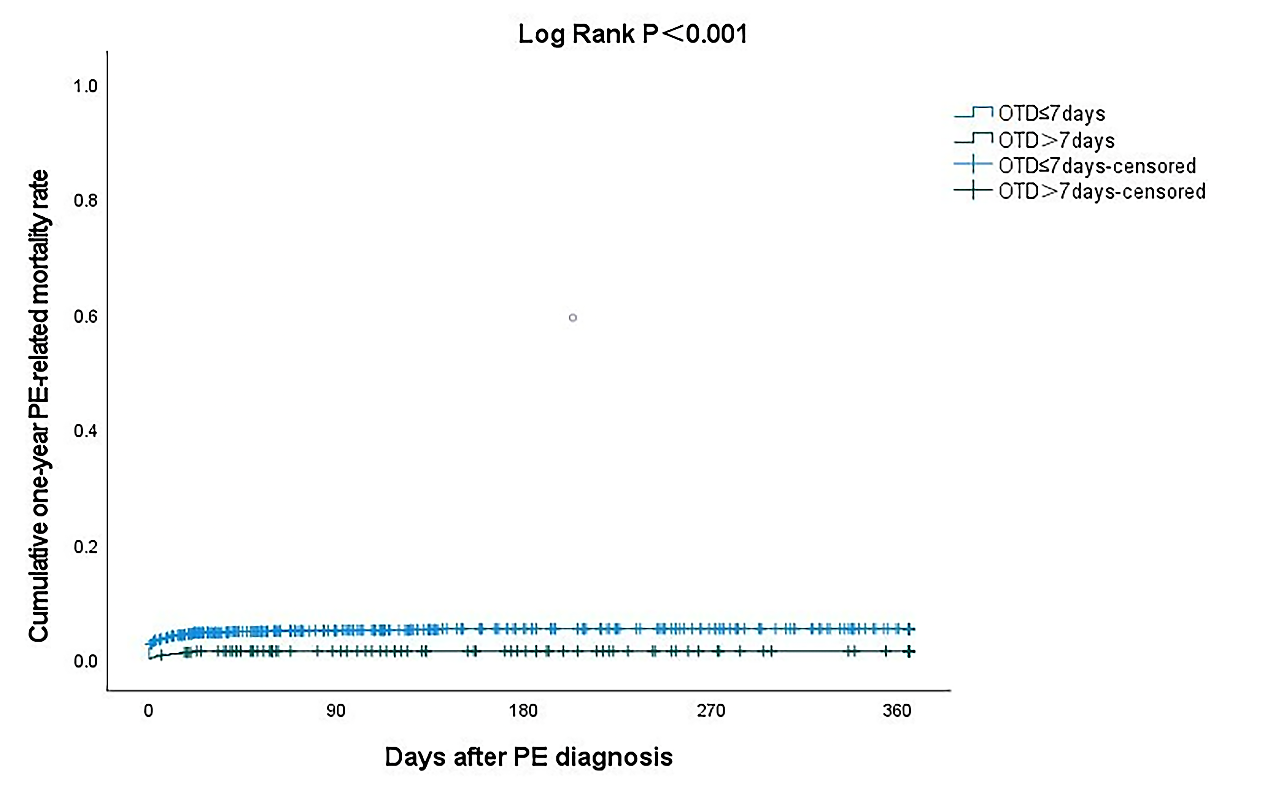
**
